# Decentralized governance and artificial intelligence policy with blockchain-based voting in federated learning

**DOI:** 10.3389/frma.2023.1035123

**Published:** 2023-02-16

**Authors:** C. Alisdair Lee, K. M. Chow, H. Anthony Chan, Daniel Pak-Kong Lun

**Affiliations:** ^1^School of Computing and Information Sciences, Caritas Institute of Higher Education, Hong Kong SAR, China; ^2^Department of Electronic and Information Engineering, The Hong Kong Polytechnic University, Hong Kong SAR, China; ^3^School of Business and Hospitality Management, Caritas Institute of Higher Education, Hong Kong SAR, China

**Keywords:** blockchain, intelligent agent risk management, Internet of Things cost-efficiency, immutable federated learning forecasting norm, EVM compatible smart contract, fruit supply chain simulation, machine learning

## Abstract

**Introduction:**

Fruit losses in the supply chain owing to improper handling and a lack of proper control are common in the industry. As losses are caused by the inefficiency of the export method, selecting the appropriate export method is a possible solution. Several organizations employ only a single strategy, which is mainly based on a first-in-first-out approach. Such a policy is easy to manage but inefficient. Given that the batch of fruits may become overripe during transportation, frontline operators do not have the authority or immediate support to change the fruit dispatching strategy. Thus, this study aims to develop a dynamic strategy simulator to determine the sequence of delivery based on forecasting information projected from probabilistic data to reduce the amount of fruit loss.

**Methods:**

The proposed method to accomplish asynchronous federated learning (FL) is based on blockchain technology and a serially interacting smart contract. In this method, each party in the chain updates its model parameters and uses a voting system to reach a consensus. This study employs blockchain technology with smart contracts to serially enable asynchronous FL, with each party in the chain updating its parameter model. A smart contract combines a global model with a voting system to reach a common consensus. Its artificial intelligence (AI) and Internet of Things engine further strengthen the support for implementing the Long Short-Term Memory (LSTM) forecasting model. Based on AI technology, a system was constructed using FL in a decentralized governance AI policy on a blockchain network platform.

**Results:**

With mangoes being selected as the category of fruit in the study, the system improves the cost-effectiveness of the fruit (mango) supply chain. In the proposed approach, the simulation outcomes show fewer mangoes lost (0.035%) and operational costs reduced.

**Discussion:**

The proposed method shows improved cost-effectiveness in the fruit supply chain through the use of AI technology and blockchain. To evaluate the effectiveness of the proposed method, an Indonesian mango supply chain business case study has been selected. The results of the Indonesian mango supply chain case study indicate the effectiveness of the proposed approach in reducing fruit loss and operational costs.

## 1. Introduction

### 1.1. Background

#### 1.1.1. Current fruit ripening problems

In the past 5 years, the majority of cargoes and shipments have employed a first-in-first-out (FIFO) allocation strategy that does not involve using sensor devices or shelf-life knowledge. Consequently, the mango market experienced significant maturation losses. As a member of the category of perishable fruits, mango has a special organoleptic characteristic that is subject to continuous change throughout supply chain activities. The problem of overripe fruit spread from upstream to downstream. Mango losses and quality problems are related to pre-harvest conditions and post-harvest management of the supply chain structure and operations (Joas et al., 2010). Such losses and quality problems have been traced back to farm operations, such as picking, washing, and drying, and logistics operations, such as packing, storing, and exporting. The majority of losses occur at the distribution stage owing to frequent handling complications, unexpected events, transportation conditions, and operational practices. In particular, when mangoes are transported over long distances and for lengthy transit periods along global supply chains, the issues of fruit loss and quality become more severe (Canali et al., 2017). Therefore, logistics and inventory management are considered the primary causes of low acceptance rates and fresh fruit losses during the retail and consumption stages (FAO., 2011). For instance, Ridolfi et al. (2018) reported that mango losses amount to 45.6% of the total production. Mango losses can be substantial, ranging from 19 to 46% of the crop. The losses incurred because of transport, storage, or over ripening in different countries are listed as follows:

- Bangladesh: 8.2% out of 25.5% (Ali et al., 2019).
- Ghana: 29.6% out of 45.6% (Ridolfi et al., 2018).
- Philippines: 3.5–4.9% out of 19.0–33.9% (Galvan, 2020).
- India: 9.6% out of 34.5% (Sab et al., 2017).
- Ethiopia: 13.4–15.7% out of 40.7% (Yeshiwas and Tadele, 2021; Tarekegn and Kelem, 2022).

Moreover, the Queensland Government (2017) found that the sources of mango losses start at the farm level and progress to the post-harvest level. Other sources of loss are in the wholesale and retail stages, primarily attributable to inadequate storage facilities and operations, low-tech packaging methods and materials used, a lack of cold chain containers and trucking facilities, and inadequate road maintenance and infrastructure networks (Albert and Barabási, 2002; Ruiz-Garcia and Lunadei, 2010). The shelf-life of mangoes can be reliably extended when stored over the supply chain cycle within the recommended temperature management ranges of the cool chamber: 10 to 12°C for storage, 12–16°C for transport, and 18–22°C for ripening (Queensland Government, 2017). However, farmers, logistics providers, warehouses, wholesalers, and retailers do not follow this practice.

#### 1.1.2. Fruit logistics functionalities

The proposed methodology collects status information about mangoes, such as export progress, maturity, temperature, and humidity. These are used to construct a scenario that enables better logistics management. The sensors detect the fruit maturity loss (Baietto and Wilson, 2015) using the “Export Progress” information, and the export progress loss is then estimated. Hence, the industry should stimulate advanced management ideas on strategy simulation and recoup a part of the sensor cost while increasing fruit quality (Tort et al., 2022). Using the “maturity” information, a mango ripeness data feed is essential to train a self-supervised deep learning model to predict shelf-life (Nordey et al., 2019). It provides deeper insight into autonomous fruit supply chain management and decision-making. Using the “Temperature and Humidity” information, the maturity datasets are categorized into different relative humidity and temperature ranges (Dutta et al., 2021). When real-time temperature and humidity are within the range of the datasets, there is a strong correlation between actual and predicted ripeness.

#### 1.1.3. Logistics prediction through federated learning

Developing a decisive model for destination selection requires a large amount of training data to produce an effective model. If all of the data are collected from a single source, the size of the dataset will be insufficient for the training process. However, if data are collected from different suppliers, there are security problems, update difficulties, and the risk of node failure. Therefore, federated learning (FL) was used in this study. FL aims to achieve the best model without exchanging data with individuals (Kuo and Ohno-Machado, 2018). To eliminate the problem of requiring a permission server for FL (Mammen, 2021), a serverless approach was used in the FL implementation. This machine-learning model derives from decentralized governance. Many parties can then use this machine learning model to develop scalable AI policy simulations.

##### 1.1.3.1. Benefits of federated learning, decentralized governance, and AI policy

In this study, federated learning and DGAP benefit the supply chain in five ways:

- Ensuring the responsible and ethical use of AI: AI systems are used in a fair, transparent, and respectful way. This can help build public trust in AI and mitigate the potential negative consequences of its use.
- Promoting economic growth and development: AI policy supports fruit saving, in addition to the development and deployment of AI technologies that drive economic growth and create new opportunities for workers and businesses.
- Protecting privacy and security: federated learning can help safeguard the privacy and security of individuals by establishing standards for the protection of personal data.
- Facilitating innovation and research: supply chain policy managers can encourage the development of innovative AI technologies and support fruit waste research to help advance the field.

Enhancing public awareness and understanding: the prediction model informs the public about the capabilities and limitations of the current supply chain system and its potential impacts on logistics. In the simulation study, the market using the LSFO policy (14.79%) is greater than the mango loss (6.91%), indicating a similarity of around 92% between the on-site case study and the simulation. This scalable simulation can help foster informed debate and decision-making about the role of AI in the supply chain.

#### 1.1.4. Blockchain-based federated learning with voting through smart contracts

##### 1.1.4.1. Benefits of blockchain in supply chain management

Blockchain technology has the potential to benefit supply chain management in several ways, including:

- Improved traceability: the blockchain can track the movement of goods through the supply chain, allowing companies to easily trace the origin and history of a product. This can help to reduce counterfeiting, ensure food safety, and improve the overall quality of products.
- Increased transparency: with blockchain, all parties involved in the AI training can access a shared and transparent record of data transactions. This can help boost the acceptance rate.
- Enhanced security: the blockchain's decentralized nature and use of cryptography make it a secure platform for storing a global AI model. This mitigates the risk of man-in-the-middle attacks.
- Increased efficiency: smart contracts in the blockchain can automate supply chain processes, such as the exchange of data, which reduces the time and cost of manual processes.
- Greater AI collaboration: the blockchain facilitates collaboration between different parties in a supply chain, allowing them to establish a consensus more efficiently and effectively.

Overall, the use of blockchain in supply chain management enhances the traceability, transparency, security, efficiency, and collaboration of processes, leading to improved trust.

##### 1.1.4.2. Blockchain technology selection

The blockchain-based federated learning aims to create an on-chain global AI model. A public blockchain may be the most suitable option because of the following reasons:

Decentralization: A public blockchain is inherently decentralized, meaning that it is not controlled by a single entity. This can be beneficial for the global AI model, as it ensures that the model is not controlled by any one party and can be accessed by many users. In contrast, a private or hybrid blockchain is generally better suited for use cases that require a high level of control and privacy. This cannot broadcast the global AI model.

Trust and transparency: The public blockchain is generally more transparent and secure than private blockchain technology, as it is open and accessible to all parties. This is important for the global AI model, as it ensures that the model is transparent and trustworthy and that all parties have equal access to it. However, the AI model needs to be accessible only to a limited number of users and also requires a high level of trust and accountability from private and hybrid blockchains.

Scalability: The public blockchain is generally more scalable than the private and hybrid blockchains, as it is designed to support a large number of operators and transactions. This is important for the global AI model because it ensures that the model can handle a large amount of data and users.

This study proposes a scalable FL method with blockchain using a common language/network and its data. Following the training session, the blockchain's ledger broadcasts its fragmented parameters to the blockchain itself. It contributes to a global model comprising a network and its parameters. An individual receiving the broadcasted parameters continues to train with new data to refine the global parameters. The results can subsequently be broadcast on the blockchain. Upon receiving the broadcasting and voting information from the blockchain, the algorithms decide whether the new parameters will be accepted and added to the global model. Voting is performed through a smart contract (Wang et al., 2018) without involving a central mediator, and the global model update is accepted or rejected according to the voting results. The global model then continues to adjust the parameters as the individuals asynchronously continue to learn from new data and broadcast the refined parameters. Each participant can thereafter simulate a virtual environment for the logistics flow scenario in the field. As Widi et al. (2021) only mentioned the beginning and ending maturity statuses, these data have been used in the reconstruction of the logistics flow. They can thereafter be used to analyze the progressive maturation of mango shelf-life in the experiment.

#### 1.1.5. Aim of this paper

In traditional logistic management, fruit loss information is collected at the beginning and end of the transportation route, and information on fruit ripeness throughout the journey is unknown. However, with a system simulating the mango allocation strategy, decision-makers can obtain recommendations and receive guidance generated with decentralized governance by artificial intelligence (AI) policy (Chen et al., 2021). The simulation promotes an impact that influences peers and forms a consensus when making policy decisions. Without export simulation, it is difficult to determine the amount of fruit wasted in the company. It is also difficult for storeroom workers to execute a single strategy to address the supply chain issue. The proposed system collects data, shares experiences, learns from quick trials, transforms wisdom into a global AI model, and broadcasts it to a permanent public network. This study aims to (1) propose scene simulation, (2) launch a permanent intelligence broadcaster, and (3) improve the cost-effectiveness of the fruit supply chain, as presented in the analysis of Indonesia's most important fruit commodities (Mahendra et al., 2008). Because the agricultural supply chain industry has to handle perishable production, planting, growing, and harvesting processes that depend on climate and season, and yields of varying shapes and sizes, such management is more complex than that for non-agricultural supply chains.

#### 1.1.6. Structure of this paper

This paper is organized as follows:

Section 2 presents a literature review of the Indonesian mango industry to study the ripeness status of mangoes in greater depth.

Section 3 describes the three-process flow-integration design and blockchain-based AI models.

Section 4 aims to fill the gap in the missing digital information with the reproduction technologies for the individual scenes, such as stochastic simulations of fruit loss.

Section 5 presents an evaluation of the simulation of the launch policy trial of an AI agent.

Section 6 discusses real-world industrial applications and the known variables that could limit the interpretability of simulation outcomes.

Section 7 summarizes our research findings.

### 1.2. Technologies checklist

The technologies used in this proposal are summarized below for ease of reference only and will be explained in subsequent sections.

In data collection:

1. Anto Wijaya Fruit: Widi studied this mango company (Widi et al., 2021).
2. Digital twin: a real-time virtual representation of the physical identity of a mango.
3. Double helix: this is a forecasting model's data feed within Deep Q-learning which simulates a management process.
4. Fractal theory: Fractals attempt to model complex processes by searching for a simple and efficient management process. Most fractals operate based on the principle of forecasting and selecting a feedback loop.
5. Interpretive structural modeling (ISM): This method transfers a flow chart design of the Anto Wijaya Fruit structure (Widi et al., 2021) to our proposed system (state diagram).
6. Tridge.com: a website that contributes maximum export value (EV) data^1^.

In AI:

1. Deep Q-learning model: a neural network maps input states to (action and Q-value) pairs, and the learning process uses two neural networks.
2. Long Short-Term Memory Model (LSTM): this network model is ideal for forecasting based on time-series data, as there may be lags of unknown duration between the ripeness events in the time series.
3. OpenAI gym: this provides numerous environments so that everyone has a common one to test the policy.
4. Model parameter: a configuration variable inside the model whose value is derived from the data. Models require parameters when making predictions. These values define the model's skills.
5. Regression method: this method generates a function curve that best represents all observations.
6. Scene construction fidelity: this is the threshold for strategy dominance during scene construction.
7. Stochastic simulation: the simulation's variables can change stochastically with their respective probabilities.

In Blockchain:

1. Blockchain network: the technical infrastructure that provides smart contract services for applications.
2. Ethereum virtual machine (EVM): a “virtual computer” that developers use to create decentralized applications (DApps) and execute and deploy smart contracts on the blockchain network.
3. EVM-compatible smart contract: a smart contract creates an EVM-like code execution environment, enabling Ethereum developers to easily migrate smart contracts to EVM-compatible chains.
4. Hive mind: when an individual has a strong tendency to fall into group decision-making *via* a smart contract by aggregating AI features.
5. Layer 1: the main blockchain network responsible for on-chain data transactions.
6. Permanent AI model broadcaster: a smart contract broadcasts model parameters permanently to allow grassroots voting to adopt a mainstream AI version as global skill-oriented programming, such as fruit shelf-life forecasting.
7. Smart contracts: programs stored on the blockchain run when predetermined conditions are met.
8. Smart contract address: a collection of codes and data residing at a specific address in the blockchain network.
9. Solidity programming language: object-oriented programming used to build and design smart contracts on a blockchain platform.

## 2. Literature review

### 2.1. Shelf-life issues

We examined the Indonesian mango industry as a case study to illustrate the current operational issues. Ali et al. designed a mini-experiment to measure the amount of wasted fruit in 2019. After an investigation of the mango industry, results revealed total postharvest losses of mangoes at different stages between harvesting and consumption (25.51%). The majority loss dominance percentage, L, was 6.91% (Ali et al., 2019) due to the lack of shelf-life information. The exploration of the primary industry led to the finding of one mango supplier (Anto Wijaya Fruit).

### 2.2. Cost issues

The purchase of Internet of Things (IoT) devices could not solely rely on a single source of funds because locals usually paid with cash after selling mangoes in the supply chain exchange (Widi et al., 2021). Increasing the budget for purchasing sensing devices was critical for transferring the sustainable balance generated by preserving mangoes before they perished. We selected the TGS 2,600 and DHT11 sensors because of their high reliability as IoT components (Song et al., 2020). TGS 2,600 will reach the end of its service life after 10–13 years (Eugster et al., 2020). Moreover, the DHT11 sensor provides precise temperature and humidity readings and ensures high reliability and long-term stability (Srivastava et al., 2018).

### 2.3. Sensor issues

Apart from the cost, another key issue was the lack of sensors to detect the shelf-life of mangoes. Thus, the retailers had only been using the FIFO strategy to reduce mango loss. However, the degree of ripeness may not correlate with the arrival order. This strategy, therefore, risked reducing the shelf-life of some fruits. Ali investigated the market dominance of mango loss when using the FIFO strategy (Ali et al., 2019). Mango failure datasets were collected between March and April 2017–2019 to examine the quality of mango cultivation in five districts: Rajshahi, Chapainawabgonj, Cuadanga, Meherpur, and Satkhira. It was assumed that all postharvest mangoes had approximately 4 weeks of shelf-life between collection and consumption by collectors, merchants, wholesale agents, retailers, and finally, end consumers. The retailer had to separate the fruits into different sizes and sell them within 2 to 3 days.

The FIFO strategy (Srinivasan et al., 2004) works as follows: when X is less than the quantity for the 1st week (Q1), the system displays the total profit, which is the target amount multiplied by the profit per unit for the 1st week (X^*^L1). Otherwise, it moves on to the next week when the overall goal is to subtract a smaller amount from the 1st week's amount than the number in the 2nd week (X–Q1 < Q2) (Mendes et al., 2020).

A recent survey reported mango losses in Indonesia. When 100% of the mangoes were subjected to the FIFO strategy, total mango loss due to grading and maturity issues was 6.91% (Ali et al., 2019), as follows:

- Farm level: 1% (sorting and grading), 0.95% (overripening or shriveling).
- Wholesale level: 1.2% (overripening or shriveling), 1% (immature or unmarketable size).
- Retailer level: 1.5% (overripening or shriveling), 0.26% (immature or unmarketable size).
- Storage level: 1% (overripening or shriveling).

Mango loss information was collected locally at the beginning and end stages. The timeline from unripe to ripe or overripe fruit during mango logistics is a missing piece of the puzzle, as there is neither a sensor nor a prediction throughout the logistics process (Widi et al., 2021).

It should be noted that even if the sensors are only deployed to diagnose the present mango status (early ripe, ripe, or overripe), scenario planning and high-dimensional simulation cannot be conducted without a strategy-making agent, which is enabled by a global forecasting model.

The upcoming session will use smart contracts to allow distributed ledger systems to reach an agreement on a global forecasting model for the AI agent. As a result, the system meets the criteria for Supply Chain 4.0.

## 3. Methods

This section discusses the design of integrated process flows in Section 3.1, with the AI and blockchain frameworks in Section 3.2. The overall system was designed to standardize the framework. The distributed features are stored in a public ledger and processed through a smart contract. The strategy-making agent can thereafter determine the optimal policy in the simulation. By transferring fruit features into an immutable code, this method is unique in promoting an economical supply chain 4.0 structure from a centralized statistical strategy into a decentralized probabilistic strategy.

### 3.1. Design of process flows integration

The proposed system contributes to a potential Supply Chain 4.0 for addressing the issues discussed in Section 2. The ISM (Pfohl et al., 2011; Astuti et al., 2014) transforms the operation flow of the “Anto Wijaya Fruit” company (Widi et al., 2021) into our design for process flows covering product, finance, and risk, as outlined below.

**Figure 1:**
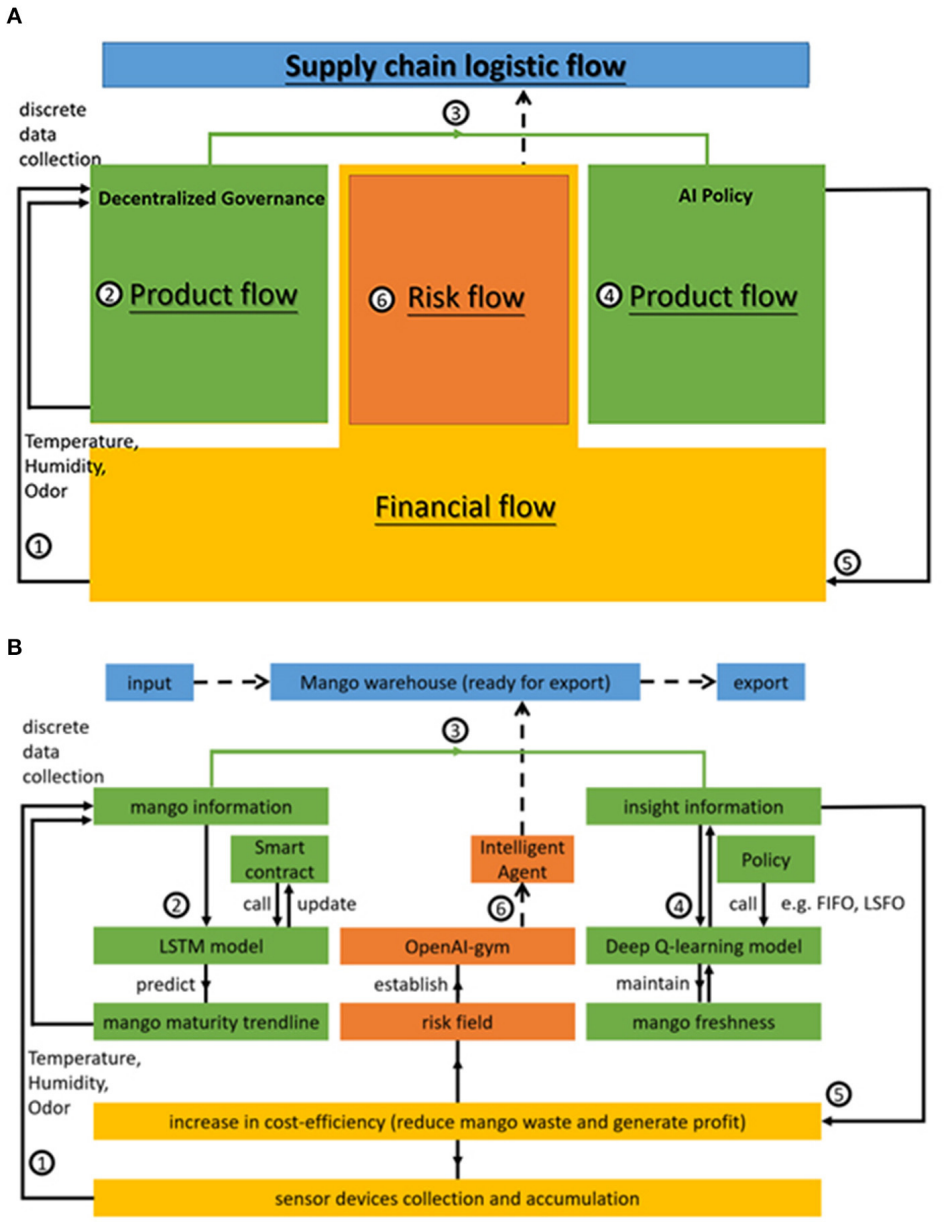
**(A)** Double-helix architecture based on AWF structure. **(B)** Double-helix architecture based on AWF structure.

- **Process flow of mango production**. Figure 1 illustrates the double-helix architecture. When farmers pick mangoes, they place them into a deployed IoT device box. The box contains the mangoes harvested at the same time. Sensing devices can thereafter collect data on temperature, humidity, and odor. These discrete data are the training materials for a local forecasting model, the LSTM. This AI model can then predict mango maturity trends to update the mango information. Its parameters can be called or uploaded through a public smart contract, combined as a global model using decentralized governance techniques, and produce probabilistic patterns. While the governance is decentralized, mango information is transferred to insight information in the AI policy. In this regard, a discussion-making agent acts based on the future relational pattern. The collector can allocate mangoes to different retailers and dealers *via* AI agent discussion. The choices of the policy function of least shelf-life, first out (LSFO), or FIFO in a stochastic simulation are identified by the trained evaluation net and the target net within the Deep Q-learning model. The intelligent agent assists the dealer and retailer in choosing which box to select and export to consumers. This can help keep mangoes fresh and improve the source of insightful information.
- **Financial flow of sensing devices**. This insight can potentially increase cost efficiency, reduce mango waste, and generate profit. Table 1 lists Indonesian mango EV sourced from tridge.com. It should be considered that US $200 per device includes all operating and maintenance costs for a sensor device over 10 years. Equation (1) evaluates the net cost efficiency (Net CE), which is the profit due to the reduction of mango loss, and the risk of decision-making caused by the AI agent to determine the yield and purchasing power of the IoT devices.

(1a) $$\begin{array}{lll} CE & = & L\%-R\%\; \end{array}$$

(1b) $$\begin{array}{lll} Net\;CE & = & CE\;-\;\frac{\;N{\;}^{*}\left(200\;USD+x\right)}{EV} \end{array}$$

**Table 1:** Indonesian mango EV sourced at tridge.com and maximum number of sensing devices (2013–2020).

**Year**	**EV USD**	EV*(L–R%)	**Budget for sensing devices (N sets)**
2013	7,330,000	503,937.5	2,290
2014	8,530,000	586,437.5	2,665
2015	19,260,000	1,324,125	6,018
2016	21,150,000	1,454,063	6,609
2017	5,310,000	365,062.5	1,659
2018	34,700,000	2,385,625	10,843
2019	44,530,000	3,061,438	13,915
2020	82,490,000	5,671,188	25,777

where Net CE denotes net cost-efficiency,

EV denotes the export value of the mango,

L denotes mango loss (%),

R denotes risk % as a result of the less-than-ideal decision to reduce mango loss,

N denotes the number of sensing devices,

Cost of each sensing device = 200 USD,

x denotes the additional cost per sensing device.

In this case, x = sensor storage cost + implementation cost + installation cost + broadband transmission cost + visualization equipment cost. The details are as follows:

1. Sensor storage costs: it is postulated that the storage size of the device is only one percent of the storage size of the mango. The logistics company may absorb the sensor storage cost as a 1% increase in storage costs.
2. Implementation costs: the fruit supplier can pay a third-party logistics provider to implement sensor logistics.
3. Installation costs: sensors are mounted on the surface of the fruit box plate to minimize labor costs associated with inserting and removing the sensors.
4. Broadband transmission costs: as less detailed, data-intensive flows are required to be transmitted over the IoT network in the ocean, data transmission can use the original ship channel, and the satellite communication fee can be minimized. On land, the inventory's data transmission method could use LoRaWAN and 5G on a decentralized wireless IoT network, the Helium blockchain network. Regular broadband transmission expenses are relatively cheap, costing US $0.00001 per every 24 bytes sent in a packet^2^. Each device transmits the signal every minute, and their cost is approximately US $ 5, that is, six times ^*^ 24 h ^*^ 365 days ^*^ US $0.00001 per transmission = US $0.5256 per year.
5. Visualization equipment costs: sensor readings can be visualized using a web-based dashboard. They can also be accessed on a personal smartphone instead of using a dedicated display device.

Assuming a total additional cost per sensing device (x) is US $20 for the sensor's life span (10 years), the total upfront cost of purchasing and operating each sensor is US $200 + US $20 = US $ 220. The profit recovered by this AI system is EV^*^(L%–R%), where L = 6.91% and R = 0.035%. This could be used as the budget for paying the initial sensor cost. The maximum number of sensors that could be purchased using this profit is listed in Table 1.

#### 3.1.1. Risk flow associated with the use of AI agents

The risk flow in Figure 1 represents the effectiveness of launching AI to select policies in an environment. Using an OpenAI-gym library, the system simulates mango allocation during the export period in a customized risk field. This library is a toolkit for reinforcement learning. It includes several benchmark problems that expose standard interfaces and compare algorithm performance (Brockman et al., 2016). The simulation system with this library can construct a scene of fruit logistics, such as the fruit loss process (loss at the farm level, loss due to transportation, loss at the wholesale level, loss during storage, loss at the retail level, loss at the consumer level, and loss during processing). In the simulation, the intelligent agent drives the reaction process. Only a small probability of mango loss may occur using the FIFO and LSFO policies, as shown in Joas et al. (2010).

### 3.2. Blockchain-based AI models

The product flow in Section 3.1 employs blockchain-based AI models. The blockchain frameworks and associated AI models are detailed below.

#### 3.2.1. LSTM AI forecasting model

The LSTM model is an artificial recurrent neural network capable of learning long-term order dependencies in data (Hochreiter and Schmidhuber, 1997). This LSTM model undergoes four main stages, in this order: preprocessing, training, testing, and evaluation. The following paragraphs explain these stages in detail: the LSTM unit comprises a cell, an input gate, an output gate, and a forget gate (Yu et al., 2019). The cell stores values over arbitrary time intervals, while the three gates regulate the flow of information into and out of the cell. The process moves from the forget gate to the input gate and then to the output gate.

The **forget gate** uses the previously mentioned hidden state and the latest input data to determine the essential information. The previous hidden state and the latest input data are fed into a neural network that uses sigmoid activation to generate a vector in which each element is between 0 and 1. The network is trained to consider irrelevant information as 0, whereas relevant information is 1. Subsequently, the values are multiplied by the previous cell state. This process ensures that irrelevant information is multiplied by 0 and has less influence later.

Next, the **input gate** determines the unique information that is implemented in the cell state after taking into account the previous hidden state and the latest input data. The tanh-activated memory neural network generates the latest vector within a range of −1 to 1 because the neural network has already learned to combine the previous hidden state and the latest input data. After incorporating the most recent data, the generated vector indicates the extent to which each component of the cell state of the network should be updated. However, because the tanh-activated neural network does not check if it is recalling recent data, the input gate, a sigmoid-activated network with an output vector ranging from 0 to 1, is used as a filter to identify the components of the vector. Then, the output of the tanh-activated memory neural network is obtained by performing a point-wise multiplication with the input gate, and this vector is added to the cell state.

Finally, the **output gate** establishes the latest hidden state based on the newly updated cell state, the previous hidden state, and the most recent input data. The output gate uses a sigmoid-activated neural network, the previous hidden state, and the latest data to output a value from 0 to 1. This procedure ensures that only essential details are stored in the latest hidden state. However, before this process, the cell state is passed through a tanh function to output a value between −1 and 1. The tanh function output is then multiplied by the output gate to receive the current hidden state.

#### 3.2.2. Immutable broadcasting blockchain framework

The proposed smart contract broadcasts LSTM AI model parameters. Discrete mango data owners can upload local model parameters globally, while other people can read them. To design an intelligent technology policy, the developer needs scale-free reinforcement learning to compute the execution timing, whether launching a FIFO or LSFO method when processing a mango export strategy (Kaelbling et al., 1996). In addition, the forecasting model and the LSTM model, which predicts the mango maturity trend (Bruckner et al., 2013), supports AI decision-making by sharing its features on the blockchain.

The value of a blockchain lies in its ability to store intelligence. The smart contract aggregation feature allows for sharing AI models without needing middlemen. Spreading machine knowledge through the hive mind platform is likely to solve one of the enormous supply chain normalization and fruit maturity consensus problems. Just as the internet allows websites to spread information, smart contracts allow the broadcasting of model parameters. After data collection by sensors, the AI model has been trained using these datasets. Subsequently, the model predicts the ripeness of the 20% trend in future projections. The prediction uses the trained intelligence to generate a meaningful equation-free model (Kevrekidis and Samaey, 2010) to measure the relative ripeness momentum. These processes allow authorized users to vote on forecasting model proposals on the blockchain, choosing whether to merge the old and new forecasting models. However, it should be noted that there is a fee for running a smart contract. Every time a smart contract is executed, a fee must be paid to the EVM for execution. This fee is paid to the nodes that help store, compute, execute, and validate smart contracts. EVM is known as the core of Ethereum, demonstrating its importance to the Fantom network (Choi et al., 2018; Kaur and Gandhi, 2020) (layer 1).

Layer 1 is a blockchain architectural term that refers to a network that provides infrastructure or consensus on projects, such as an event-based coffee supply chain (Bager et al., 2022). A virtual machine (VM) is a computer system with complete hardware functions simulated by software and running in a completely isolated environment. By generating a new virtual image of the existing operating system, the VM performs the same functions as the Windows system; however, it runs independently from a Windows system. As the name suggests, the EVM is Ethereum's VM. Notably, there are no VMs in the Bitcoin blockchain (Nakamoto, 2008). The primary function of Bitcoin is to store data in a distributed manner, and we can record, verify, store, and replicate transaction data in this network. Ethereum is a “decentralized real-world computer,” and developers can also build DApps on it, implying that Ethereum not only needs to be able to distribute data storage but also needs to run code and conduct consensus communication (Tikhomirov, 2017; Hildenbrandt et al., 2018). If an account wants to execute a smart contract, the transfer will be completed according to the smart contract, and the relevant execution rules will be recorded in the data to guide the contract's operation. The network nodes execute the smart contract code every time the described transaction occurs through the EVM.

#### 3.2.3. Deep Q-learning AI decision model

An AI agent selects the best action for the batches of mangoes based on the estimated shelf-life or the first import's mango. The program starts by defining the parameters of Deep Q-learning, and thereafter defines three classes and a function. These three classes define the environment, neural network, and Deep Q-learning, respectively, while a function runs on the main program. The parameters for Deep Q-learning are as follows: 0.9 for Epsilon, 0.9 for Gamma, 0.01 is the learning rate in an Adam optimizer, memory capacity is 3,000, Q-Network iterations are 100, batch size is 32, and episodes are 1,000. The environment class selects a random integer between 0 and 1,200 for the shelf-life state, 480 array shapes for the shelf-life future projection and creates a store state from 1 to 480 to determine the reward. After 480 steps, the environment is reset to its original parameters and returns an array from the shelf-life state, future projection, and storage state.

## 4. Simulation process

This section explains how to operate simulated frameworks to predict and make decisions based on complete process flows.

### 4.1. “Mango digital twin” data collection and preprocessing configuration

**Figure 2:**
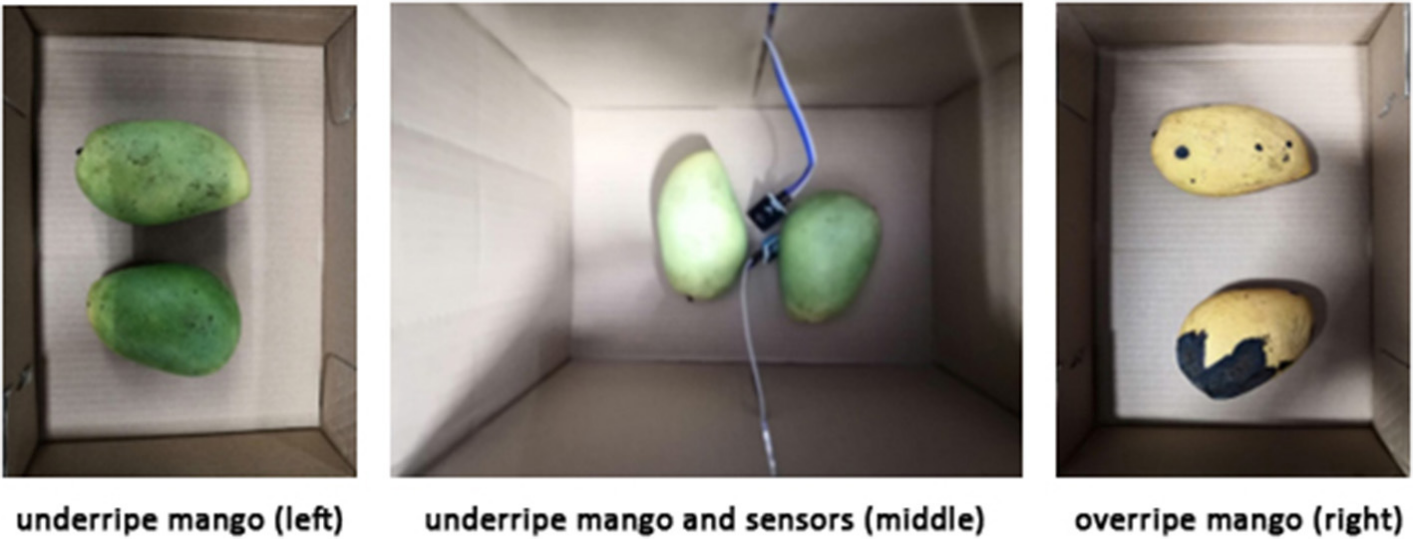
Underripe mangoes **(left)**, and overripe mangoes **(right)**.

The state of physical entities on an information platform relies on digital twin technology. Figure 2 shows two underripe mangoes inside an A4 paper box equipped with TGS 2,600 and DHT11 sensors. A Raspberry Pi collects data from these two sensors. The box remained closed for 6 days until the odor reading rose from 3 (underripe) to 7.5 (overripe).

The collected data are discrete and extensive. The machine first uses the *iloc* function to rescale the original dataset into 2,400 sampling points and process a regression method. The shelf-life of mangoes (from underripe to overripe) is irreversible. Because the reading includes the fluctuation property, it leads to an irregular shifting up and down in the shelf-life level. Therefore, a maximum function is added to address this issue by comparing the back-and-forth difference between the two frames each time.

In the regression stage, a data frame is created for odor data. It is defined using a Gaussian process model (Kocijan et al., 2004; Azman and Kocijan, 2006). The likelihood and model are initialized, and the optimal model hyperparameter is determined. The Adam optimizer uses the gaussian likelihood parameters (Rafi et al., 2016) and runs 40 training iterations. Following that, the model and likelihood are evaluated. The predictions can then be made by feeding the model through likelihood. For instance, a data frame and graph are created using data after regression. In the feature selection stage, 2,400 sample points are taken from the data frame and converted into a new one.

### 4.2. Forecasting process

The proposed LSTM neural network has a feature size of one hidden unit, one output, and one layer of LSTM to stack. A small odor value is made into a graph that represents the early ripeness of the fruit. The odor dataset is resized to 2,400 frames. This dataset is used to produce another graph that plots a portion of the original dataset. Subsequently, 80% of the data from the dataset is used for training, whereas 20% is used for testing. The training data are divided into five batches. The dataset is used in the LSTM model, which has one feature size, 16 hidden units, and a maximum epoch of 10,000. After the training stage, the testing stage begins by switching the LSTM model to the testing model. The prediction on the test dataset is made by setting the batch and feature sizes to 1. When the testing section is finished, it is plotted on a graph, and a root-mean-square deviation (RSME) is provided. Finally, the local LSTM model parameters broadcast their application to a public network in four steps:

1. Upload to an EVM-compatible smart contract on the Fantom network.
2. Interact with a global model.
3. Group into a global model.
4. Generate a shelf-life future projection for deep Q-Learning.

### 4.3. Immutable AI model broadcasting process

Several local models can be grouped into global ones through voting. In terms of the feature aggregator (FL), the voting system presents an EVM-compatible smart contract program for FL. *Voting* is a program that runs on the blockchain. This allows authorized voters to vote for proposals when they fulfill the program conditions. A solidity voting contract has two structures: a constructor and six functions. It also includes some lines of code that store the chairperson's address, the voter's address, and proposals. The following paragraphs explain voting contracts in detail.

The proposed smart contract, named “model parameter,” is a collection of functions and LSTM model information (its state). A deployed contract resides at a specific address on the Fantom network, which is an EVM-compatible blockchain. This smart contract is divided into two parts: the constructor and the function. Similar to several class-based object-oriented languages, a constructor is a special function that is executed only when a contract is created and is used to initialize the contract's data. A public function accepts a string argument and updates the “parameters” storage variable. State variables, or “parameters,” are variables whose values are stored permanently in the contract storage. The keyword “public” makes the variable available outside of the contract and creates a function that other contracts or clients can use to access the LSTM model parameters for simulation initiation.

The contract has two structures: “voter” and “proposal.” In the “voter” structure, the voter can change their choice. To ballot the proposal, each voter has votes attributed to their unique address. The “proposal” structure stores the name of the proposal and the number of accumulated votes. These structures help store vital information about voters and proposals. The program has a constructor that runs once a ballot is launched. The constructor assigns voting rights to the chairperson. Following that, it takes the address name of a proposal in bytes-32 form and initializes the proposals with zero votes. Then, it can add proposals to an array in a voting contract. Such a smart contract contains six functions, namely “giveRightToVote,” “delegate,” “vote,” “winningProposal,” “getAllProposals,” and “winnerName.” These functions are developed as follows:

1. The “giveRightToVote” function authorizes the voters the right to vote for proposals in the contract. Adding the voter's address only provides them with the right to vote once. This function can only run if the chairperson is allowed to deliver the right to vote.
2. The “delegate” function allows voters to appoint their votes to other voters by taking in the delegates' address and authorizing them to vote. This function starts by assigning the original voter's address as a reference. The function can then check two cases: (1) whether the original voter has already voted, and (2) whether the voter is self-delegating their vote. If either of these conditions is met, this function will not be executed. On the other hand, the function executes a while loop to obtain its address and to check whether the original delegate has entrusted their vote to someone else. If the original delegate's address passes the trial, it is adopted. Next, the loop function checks whether the intended delegate has assigned their vote to the delegator. If true, the function will not run to prevent an infinite loop from forming. Once the reading scan is completed, the function requests that the ledger provide its address. After receiving the delegate's address, the function sets the delegator's voting status as “voted” and stores the delegate's address in the delegator's “voter” structure. The function then checks if the delegate has already voted. If this is the case, the function would increase the proposal's vote count by one. If not, the function adds one to the number of votes to allow the delegate to vote.
3. The “vote” function lets a voter vote for a proposal by taking in the proposal's index number from the proposal array and then adding a vote to the proposal. It begins by assigning the voter's address as a reference. Then, it checks whether users have the right to vote and whether they have already voted. If either of these is true, then the function returns. Only if they pass through this check can the function store their voting status as true and write the index of the proposal into the voter's “voter” structure. It then adds a vote to the voter's total by increasing the count by one.
4. The “winningProposal” function works by finding the proposal with the highest vote count. It starts by setting the winningVoteCount variable to zero. The function can then pass the array of proposals using a for-loop. This function continues until the proposal with the highest vote count is obtained. Each loop checks whether the vote count of the proposal is larger than the variable “winningVoteCount.” If it is true, then the function sets the proposal as the winning proposal. Once the winning proposal is found, its index is returned.
5. The “getAllProposals” function passes the array of proposals and returns them all.
6. The “winnerName” function returns the winning proposal's name in bytes-32 form by calling the relevant function.

### 4.4. Simulation process

**Figure 3:**
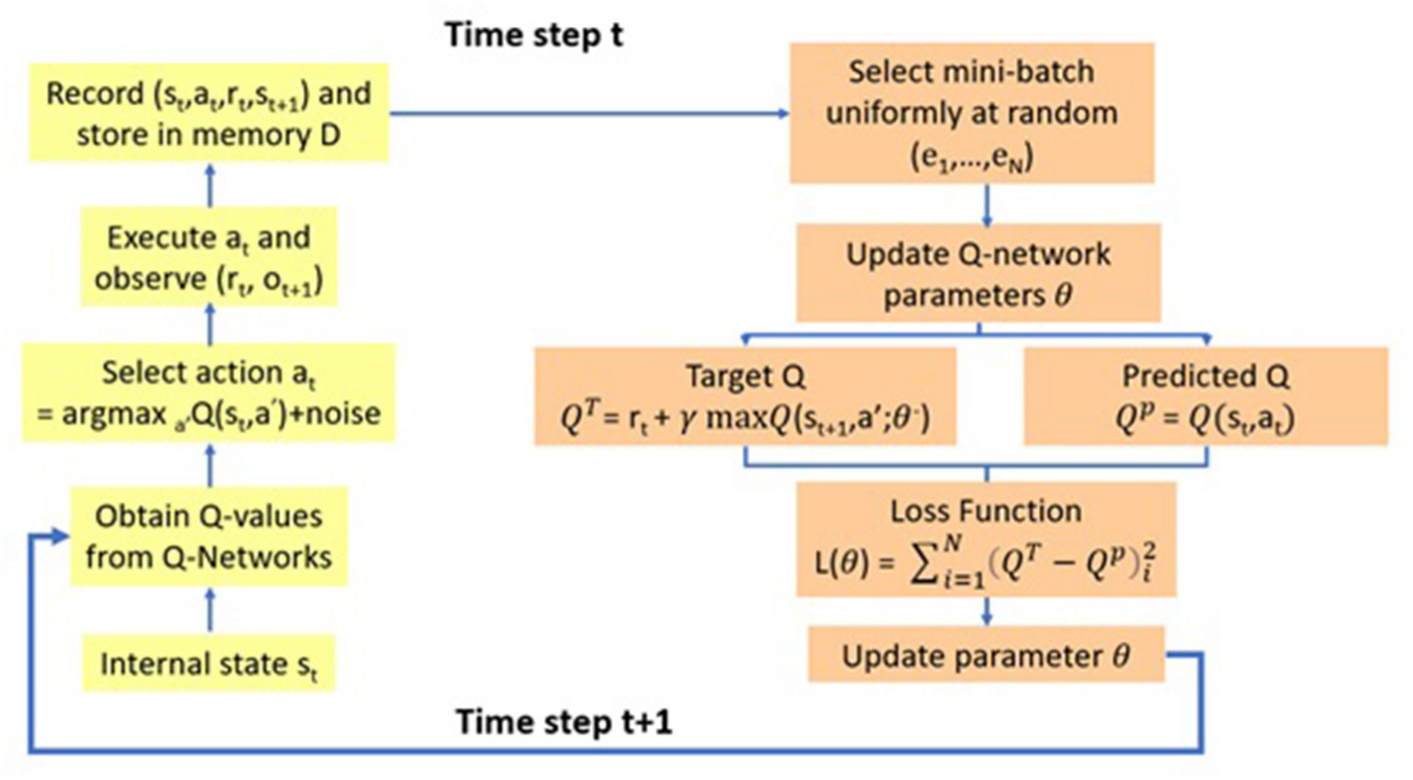
Deep Q-learning model.

**Figure 4:**
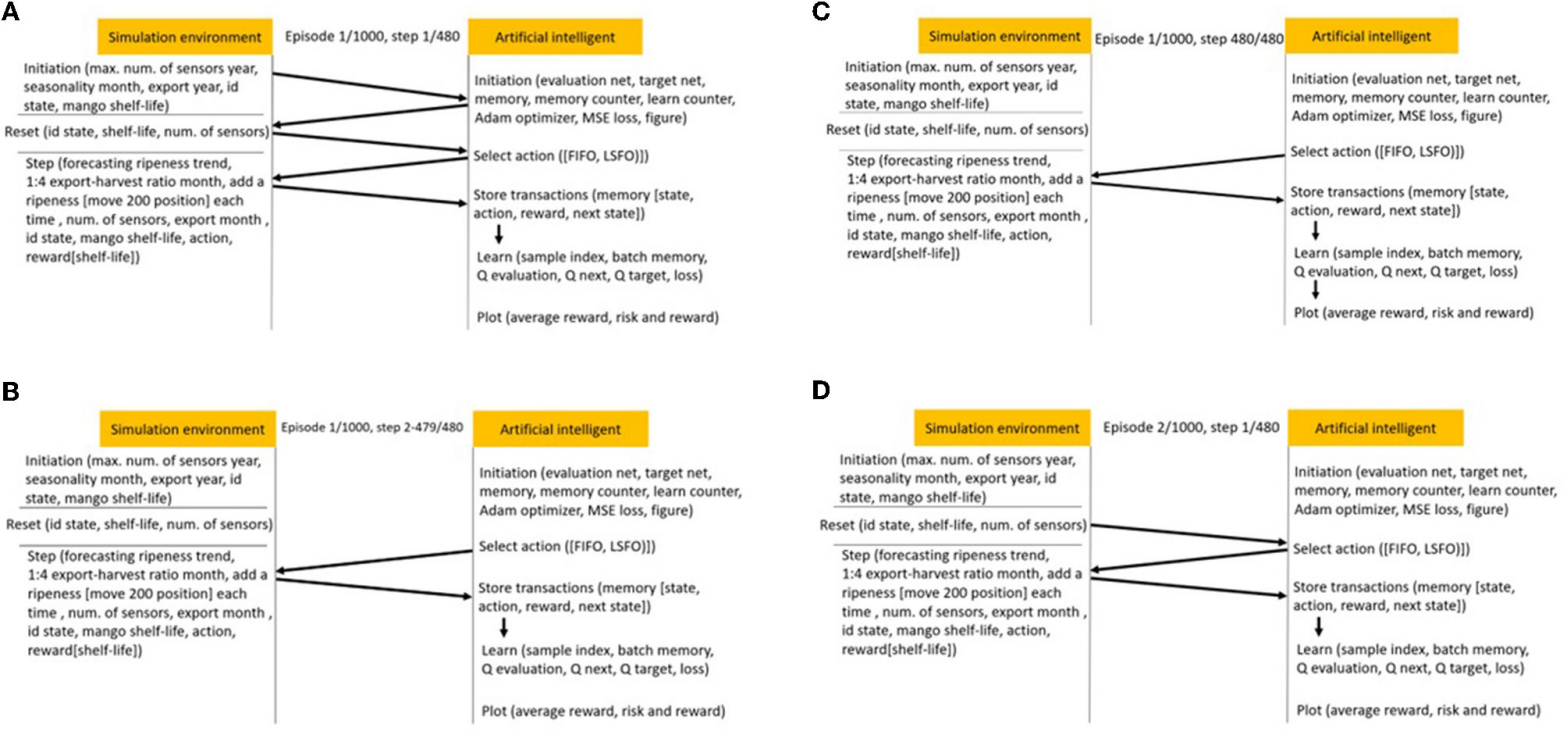
**(A)** Simulation flow in episode 1, step 1. **(B)** Simulation flow in episode 1, steps 2–479. **(C)** Simulation flow in episode 1, step 480. **(D)** Simulation flow in episode 2, step 1.

**Figure 5:**
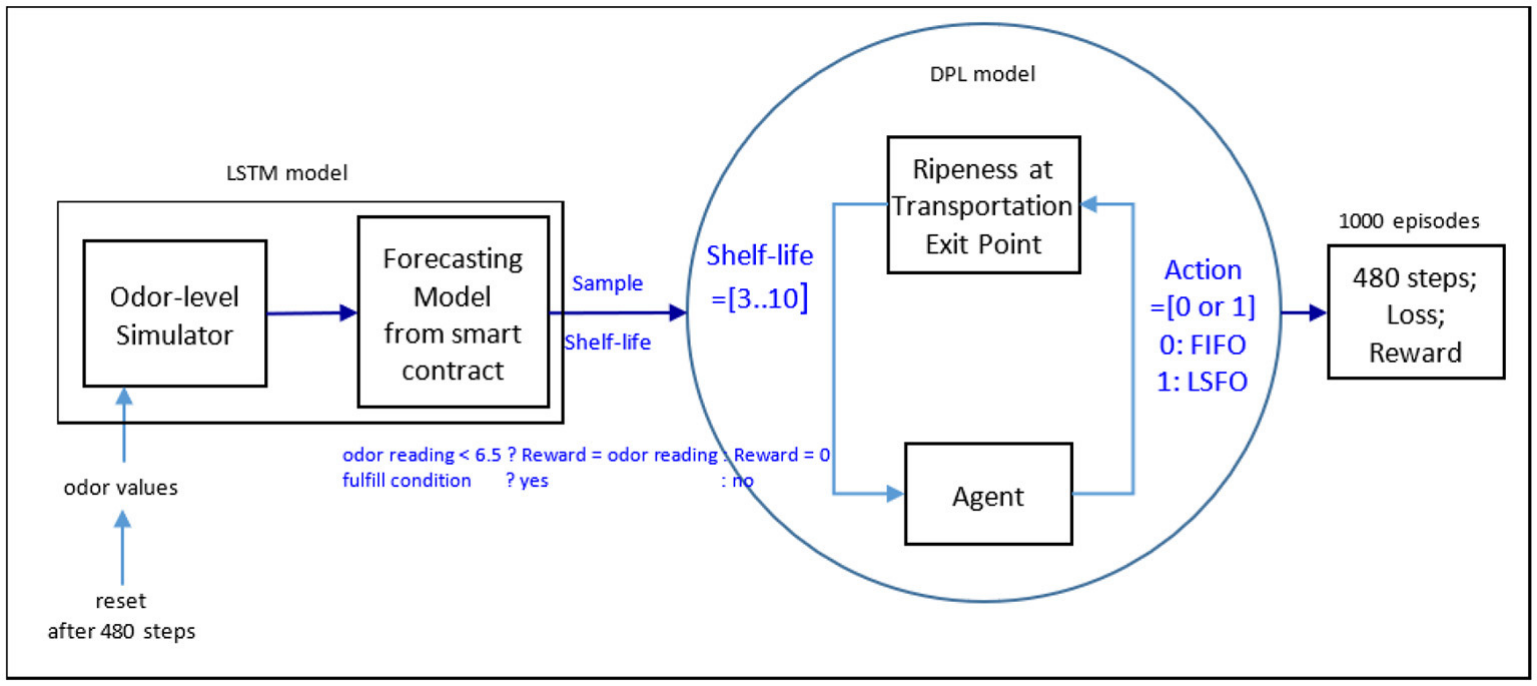
Deep Q-learning with simulated forecast scenarios.

Here, forecasting and broadcasting processes facilitate the development of a general simulation structure. OpenAI-gym is a simulation structure package that includes three simulation initiation functions: (1) definition, (2) step, and (3) reset. The program begins by defining the parameters of a Deep Q-learning model before launching three classes and a function. The first step of the main program defined three classes (environment, neural network, and Deep Q-learning). The following paragraphs explain the program in detail, as shown in Figures 3–5. In the internal state *s*_*t*_, the shelf-life is determined by the odor of the fruits, which reflects the ripeness of the batch of samples. The Q-values from Q-Networks can then be used to select an action to execute and observe the outcome.

Because the study collected 8 years of EV data, the machine used those and calculated the total steps for each episode, that is, (4 harvesting times + 1 delivery) × 12 months × 8 years = 480 steps. The deep Q-learning class starts by defining a “memory counter” as zero and a “learn counter” as zero. The deep Q-learning class has a function known as “store trans,” which counts and stores a state *s*_*t*_, an action *a*_*t*_, a reward *r*_*t*_, and a next state *s*_*t*+1_ in memory *D*. It also has a function known as “choose action,” which determines whether to pick the new or old model. Another function in the class is the “plot” function, which charts a graph of total reward against episode.

In addition to the memory counter and the choosing action, this class has another function known as “learn.” This function is called 1000 times before updating the target network. After 100 episodes, the memory can then provide an experience to help select an action in the remaining 900 episodes of the simulation. Memory has four sections: state, action, reward, and next state. Within the memory capacity, the memory counter follows a step counter to select an action based on finding the largest action value in the observation space (shelf-life future projection and storage state). When the agent selects the action value in the shelf-life future projection, it chooses to execute the LSFO policy. Alternatively, there is an option that uses the FIFO strategy after receiving an action index. The AI agent experiences each learning process to determine an optimal strategy.

In every learning cycle, the learn counter follows the step counter to obtain batch memory. In each episode, a new data frame is created by passing the odor data frame through the reset function of Deep Q-learning. When the step counter is zero, the “main” function also creates memory slots to concatenate the data from the array created by the Deep Q-learning class. The memory slot is then passed to the “choose action” function for digit-field construction.

The digit-field construction method is dependent on the prepared model parameters and logic. Figure 4 illustrates the AI agent being trained inside the digit field after having processed 1,000 episodes. In the first episode and first step, there are six segments in the simulation flow: initiation, reset, select action, step, store transactions, and learn. In the simulation environment, the initiation program sets up the maximum number of sensors, the seasonality month, the export year, the boxes' ID state, and the mango shelf-life for mango information. The evaluation net, target net, memory, memory counter, learn counter, Adam optimizer, mean-square error loss, and output figures are all machine learning settings that are part of the AI initiation program. In the simulation environment, the reset program resets the box ID state, fruit shelf-life, and number of sensors. The AI agent can then obtain the information necessary to select an action (FIFO or LSFO) and execute it in the simulation step program. The export-harvest ratio per month was assumed to be 1:4 for mango box allocation in the warehouse. Before making a decision, the ripeness sampling position of the received boxes was increased by 200 steps to simulate the ripeness speed while maintaining the testing sampling position for the forecasting model to predict mango shelf-life. The program eliminates the box ID to simulate export scenarios. The AI agent can then memorize rewards based on the mango shelf-life of exported boxes and store transactions in its memory to learn from the experience gained in this step.

The AI agent only processes two segments between two and 479 steps in the first episode: (1) select an action to respond to the step function, and (2) store transactions from the step function. At the first episode and 480^th^ step, the simulation process is the same as it was at the first episode and first step, and the average reward, risk, and reward are plotted. In the second episode and first step, the simulation environment needs to be reset before the AI agent's learning process can continue.

Figure 3 describes the Deep Q model, which integrates deep learning and Q-learning (Fan et al., 2020), and selects a mini-batch uniformly at random to update the Q-network parameter θ. The model can thereafter operate two neural networks to map the input state to action and Q-value pairs, using the same architecture but different weights. The two networks are the Q and the target networks. The target network is identical to the Q network, whereas the Q network is trained to produce the optimal state-action value. In an arbitrary number of steps, the network function replicates the weights from the Q-network to the target network. In addition to the two neural networks, it also has a component known as “Experience Replay.”

Experience Replay interacts with the environment, which gathers a training sample saved as training data. The function is performed by selecting an ε-greedy action from the current state and executing it in the environment that receives a reward and the next state. It can store the observations as a sample of the training data.

In the following step, the Q and target networks are used to predict a projected Q value and a target Q value, respectively. The function is executed by taking a random group of samples from the training set and inputting them into the target and Q networks. The Q network predicts the Q value for the action by combining the current state and action of each data sample to obtain the predicted Q value, whereas the target network predicts the target Q value by taking the next state from each data sample to compute the best Q value of all possible actions that could have been taken from the state. The target Q value then becomes the target network output plus the reward.

After defining the predicted Q value and the target Q value, the difference between the two is used to determine the mean squared error loss. In the loss function, gradient descent can then be used to back-propagate the loss and update the parameter θ of the Q network. The gradient descent concludes the processing for time step t. However, no loss or back-propagation is computed for the target network because it is not trained. In the next time step, t + 1, the processing is repeated, which allows the Q network to learn to predict more accurate Q values, while the target Q values are temporarily maintained. After an arbitrary number of steps, the weights of the Q network are copied to the target network, assisting the target network in receiving the improved weights to predict more accurate Q values. The processing can then resume as before.

### 4.5. Bottlenecks

This section explains the bottlenecks of the simulation and Deep Q-learning/deep policy learning program. In designing the recurrent run simulation decision, the study encountered two bottlenecks.

First, the number of exports is limited to 30 per month. If $\frac{1}{30}$ of the goods are shipped every day, 30 decisions must be made per month, and the upper limit of the number of decisions is equal to the required number of sensor devices. Furthermore, if there is only one harvested mango box per shipment, the decision between FIFO or LSFO must default to FIFO due to the absence of multiple harvested mango boxes in the shipment. The shipment system has no alternative option to choose the export sequence based on shelf-life in this scenario.

Second, the mango shelf-life record is 6 days, which is equal to the initial value; thus, the first 3 days would be the random range for initializing the value and the next 3 days would be the buffer for waiting for export activity.

In the simulation, the initialization function is set as four times per month for harvesting and then shipping once a month, with an interval of 0.5 days for each harvest, as listed in Table 2.

**Table 2:** Mango shelf-life in an export cycle.

**Token ID**	**1^st^ harvest**	**2^nd^ harvest**	**3^rd^ harvest**	**4^th^ harvest**	**Export 1**
	**Step 1**	**Step 2**	**Step 3**	**Step 4**	**Step 5**
1	+0.5 day	+1 day	+1.5 days	+2 days	Reset
2		+0.5 day	+1 day	+1.5 days	Reset
3			+0.5 day	+1 day	Reset
4				+0.5 day	Reset

Based on fractal theory (Higuchi, 1988), the morphology of mango ripening distribution is self-similar in every export cycle. The local shape is similar to the overall state of a fractal supply chain (Nishiguchi and Beaudet, 2000). Because the overall signal shape and the two subdivided signal shapes are self-similar in appearance, using one sensing device to collect the maturity of the harvest is equivalent to using two sensing devices or more at each harvest, because the collected mangoes are 100% shipped. As a result, the minimum number of sensing devices required per month is equal to the number of harvests per month. The AI supports distribution management by taking strategic actions to ship fresh fruit batches to destinations at different distances to minimize overall loss. In this system, the forecasting model and reinforcement mechanism are used to derive optimal strategies for allocating batches of fresh fruits to export destinations, such that minimum spoilage and higher efficiency can be achieved. Based on the current agent brain ram information (shelf-life position, shelf-life future projection, store state, action, reward, next shelf-life position, next shelf-life future projection, and next store state), the agent determines the best destinations to ship the batches of fruits, as expressed in Algorithm 1.

**Algorithm 1:**
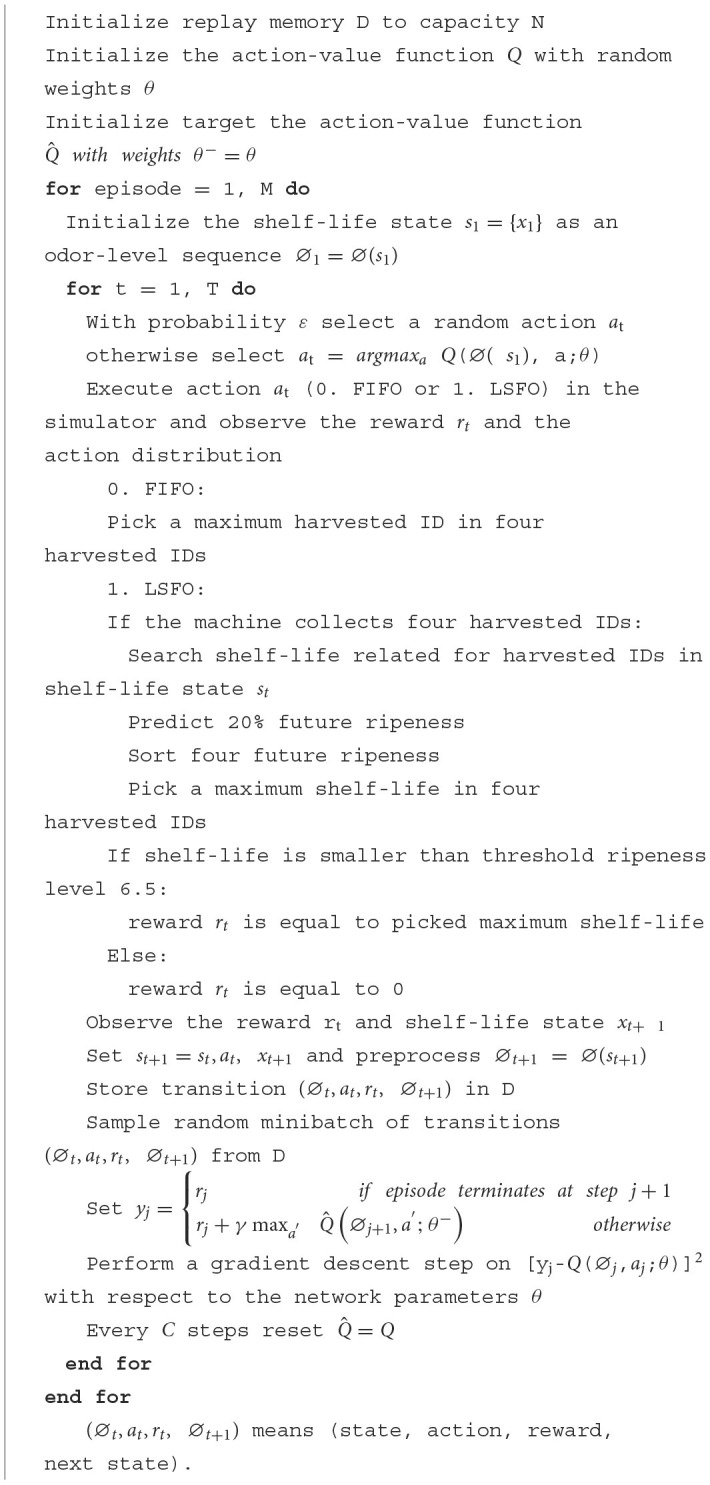
Deep learning pseudocode.

## 5. Simulation results

**Figure 6:**
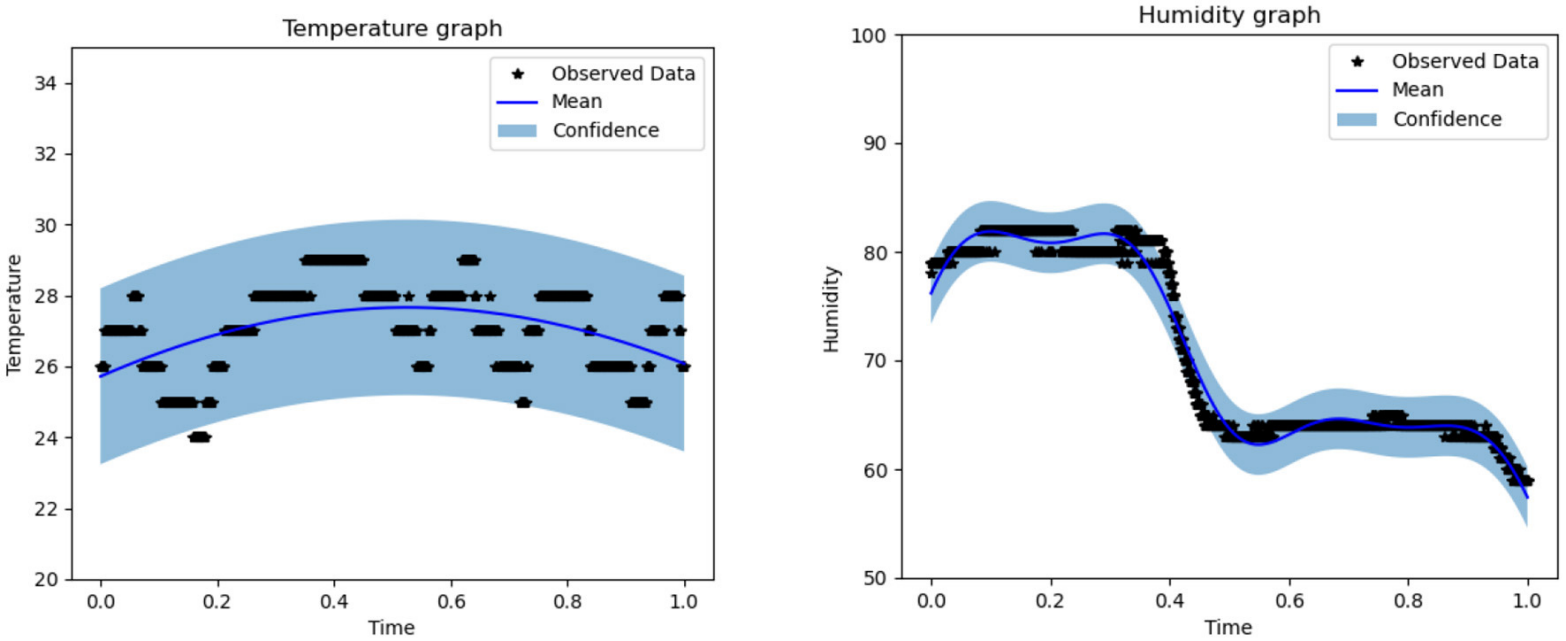
Temperature **(left)** and humidity **(right)** boundaries for the forecasting model.

The fundamental idea behind the fruit supply chain simulation is to estimate the fidelity of mango maturity loss. Because of the environmental constraints listed in Section 6, such as temperature and humidity, the outcome of the data-driven experiment can be limited. The odor pattern is valid only when the temperature and humidity are within the recorded data boundary. Temperature data were subjected to 40 iterations. Throughout these, the temperature data experienced a decrease in loss from 2.484 to 1.721, an increase in the length scale from 0.693 to 1.384, and an increase in noise from 0.693 to 1.541, as shown in Figure 6. Temperature data also showed maximum and minimum values of 30.1385° and 23.2405°, respectively. The humidity data were also iterated 40 times while experiencing a decrease in loss from 11.106 to 2.766, a decrease in length scale from 0.693 to 0.152, and an increase in noise from 0.693 to 1.939, with maximum and minimum values of 84.6541 and 54.6122%, respectively, as shown in Figure 6.

**Figure 7:**
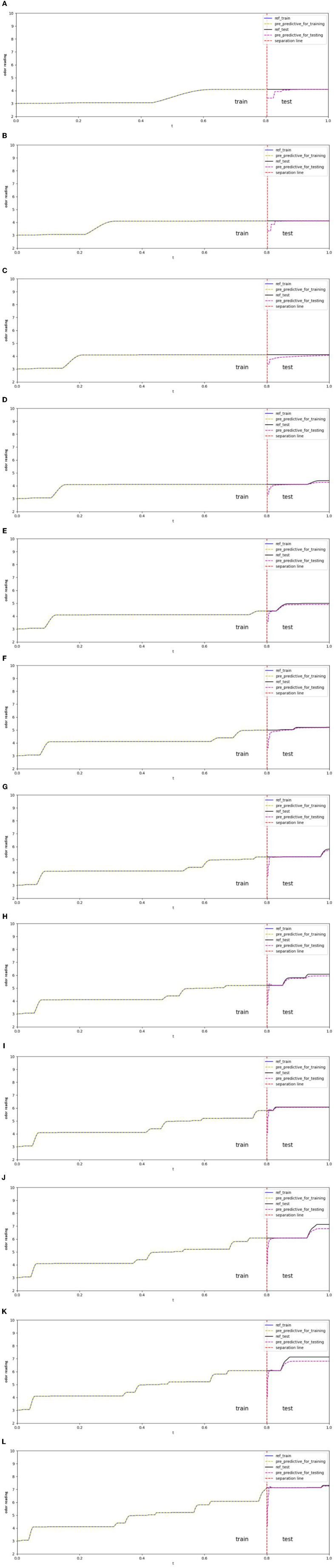
**(A)** Total of 200 odor-sampling points in the forecasting model. **(B)** Total of 400 odor-sampling points in the forecasting model. **(C)** Total of 600 odor-sampling points in the forecasting model. **(D)** Total of 800 odor-sampling points in the forecasting model. **(E)** Total of 1,000 odor-sampling points in the forecasting model. **(F)** Total of 1,200 odor-sampling points in the forecasting model. **(G)** Total of 1,400 odor-sampling points in the forecasting model. **(H)** Total of 1,600 odor-sampling points in the forecasting model. **(I)** Total of 1,800 odor-sampling points in the forecasting model. **(J)** Total of 2,000 odor sampling points in the forecasting model. **(K)** Total of 2,200 odor sampling points in the forecasting model. **(L)** Total of 2,400 odor-sampling points in the forecasting model.

In Figure 7, the shelf-life future projection uses 12 different sample dataset sizes ranging from 200 to 2,400 frames, with an increase of 200 frames each time. The shelf-life future projection maintained a loss of 1e-5. All projections contained 80% of the data for training and 20% for testing. In Table 3, the readings for the epoch have maximum, minimum, and average values of 7,698, 3,528, and 5,284.667, respectively. The test scores of the root mean square equation (RMSE) have maximum, minimum, and average values of 0.35, 0.15, and 0.2367, respectively. In the 200, 400, and 800 datasets, the graph reached a plateau at approximately four readings. In the 1,000 datasets, the graph reached a plateau at approximately 4.3 readings. The graph reaches a plateau at approximately five readings for the 1,200, 1,400, and 1,600 datasets. In the 1,800, 2,000, and 2,200 datasets, the graph reached a plateau at approximately six readings. In the 2,400 datasets, the graph reached a plateau at approximately seven readings.

**Table 3:** Test score RMSE and epoch for forecasting.

**Dataset[:N] maintains loss at 1e-5**		
**N**	**Epoch**	**Test RMSE scores**
200	4,153	0.25
400	6,368	0.2
600	6,863	0.22
800	6,155	0.17
1,000	4,885	0.15
1,200	4,250	0.23
1,400	7,698	0.21
1,600	5,619	0.21
1,800	3,775	0.21
2,000	5,310	0.3
2,200	4,812	0.35
2,400	3,528	0.34

The cost-efficiency of purchasing IoT devices is based on data from the past 8 years (2013–2020). Risk data were collected after processing 1,000 episodes. When the forecasted odor was greater than 6.5, implying that the mango inside the box was overripe, the reward was counted as 0. The ultimate reinforcement model reward was then generated.

**Figure 8:**
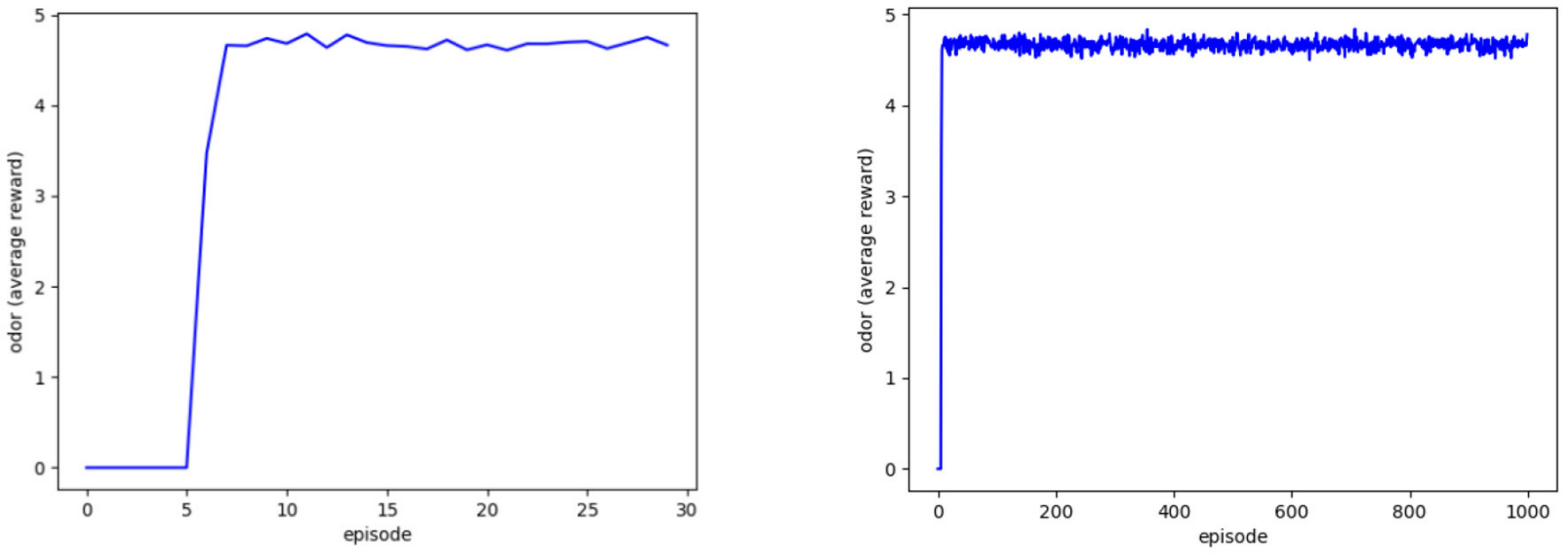
Agent's average reward at 30 **(left)** and 1,000 episodes **(right)**.

The Deep Q-learning model program aims to output the distribution of selecting FIFO and LSFO policies, such that mango ripeness can maintain freshness starting from seven episodes. Figure 8 shows the average rewards for readings ranging from 3 to 6.5. This method can ideally reduce the loss from 6.91 to 0%. The reward, on the other hand, is sampled in the last step of each episode, resulting in five overripe signals within a 1,000-time window. The simulated output concluded that the simulated loss improvement was adjusted from 6.91% (loss percentage, L%) to 0.035% (risk percentage, R%), as expressed in equation 2:6.91% × 5/1000 = 0.035% (Canali et al., 2017).

The reference-based mango market has 93.09% market dominance, 100% FIFO strategy, and 6.91% of mangoes lost owing to an unknown shelf-life. However, a simulated policy coverage is proposed in which 85.21% of the market uses the FIFO strategy while 14.79% uses the LSFO strategy.

These strategies had a tolerance of approximately 8% in terms of scene construction fidelity. This LSFO policy would also require 100% of the sensors to be used for the LSFO strategy, whereas no sensors would be required for the FIFO strategy. A total of 100% of the sensors indicate the proper number of sensors, which is equal to the number of harvesting times according to fractal theory.

## 6. Discussion

### 6.1. Discussion of results

The proposed method can contribute to the fruit industry through more accurate estimation of shelf-life and better recommendations on the delivery sequence of product batches to their destinations. Mangoes exported from Australia go through a long journey before arriving at destinations in Southeast Asia, such as Indonesia, Singapore, and Hong Kong. Mangoes are highly sensitive to various environmental conditions. For instance, maintaining the mango fruit at 12°C through the supply chain may help achieve a longer shelf-life, whereas increasing the average holding temperature to more than 20°C may shorten the shelf life. If the mangoes are delivered to the retailers within the right period of time, it helps increase the sales of the retailer and, thus, the returns of the growers. Because different batches of mangoes may exhibit different physical environments after harvesting, the deployment of sensor devices to track their ripeness provides invaluable information for decision-making in the logistics management process. Obtaining fruit ripeness information may help to make better logistics decisions so that a maximum number of fruits are delivered to the destinations within their expected shelf-life.

The proposed system affects post-harvesting management and logistics management. First, the ripeness data for each batch of mangoes are collected using sensors after harvesting. Based on the ripeness information and algorithm of the AI engine, recommendations based on the forecasting model are made for shipping to the best locations among the orders. Throughout the transportation journey, more data on mango batches are collected through the internet. At the destination, the resulting shelf-life information is used as input for the reinforcement to enhance the system's performance. From a financial perspective, the initial investment in the system mainly falls on the purchase of sensors. These sensors can be reused, with an estimated lifetime of approximately 10 years. These sensor modules are designed such that they can be plugged into delivery crates or boxes, and the installation and maintenance costs are negligible compared to the value of the batch of mangoes. It is estimated that the cost should break even after shipping a large volume of mangoes.

Because local AI models are uploaded to the blockchain, the system algorithm can be further enhanced with data from individual growers being used in FL. With the employment of the proposed system, it is expected that mango loss can be reduced to less than 10%. However, the performance of the system can be further enhanced as the number of growers increases and, thus, the size of the training dataset also increases.

### 6.2. Applications for real-world industries

Supply chain 4.0 has been one of the most relevant research topics recently studied by the academic community in the field of operations management (Frederico, 2021). Decisions made throughout the supply chain may be driven by actors in the supply chain itself or by policy interventions to attract strategic production (Barbieri et al., 2020). Digitization facilitates new design changes, efficient production scheduling, smart manufacturing, and the unlimited, on-time delivery of quality products (Kumar et al., 2022). When a global forecasting model is launched, it can significantly impact the fruit storage simulation system, which is employed to establish a controllable fruit allocation method for supply chain intelligence. For instance, real-world content applications would require the user to initialize export frequency and fruit ripeness tipping points. The system contributes to the management of visualizing the current process and modifying it before taking real action, which could save enormous costs and time for the trials. Subsequently, organizations can put their plans into action, and the AI agent can provide a suitable option (an “action value”) from their action space to guide the users in distinguishing the number of mango boxes in the storage room that should be exported first.

### 6.3. Study limitations

#### 6.3.1. Methods

Successful storage of mangoes for 2–3 weeks at 12–13°C can be achieved through refrigeration (Singh and Zaharah, 2015); however, the lack of shelf-life categorization in the training model results in a shorter storage duration. The simultaneous ripening of different fruit boxes during storage hinders the frequency of fruit exports.

#### 6.3.2. Data collection

The fewer the datasets, the lower the interpretability of the real scenes. AI decision-making in the fruit supply chain is based on shelf-life. Our trained AI model feature is only available to forecast mango shelf-life from 23.240 to 30.1385°C.

#### 6.3.3. Data type

The research only has a single environmental range for mango odor data collection, which would be beneficial if more scenarios could be considered. Therefore, the actual processes involved in managing a supply chain may require more than one theoretical approach. The study could be improved by increasing the sample size at different temperatures and humidity levels so that the outcome could provide a more balanced view of the mango export process.

#### 6.3.4. Results

Environmental factors are the primary limitation of generalizing these results. The known and theoretical confounds may have been caused by the insufficient precision of the timeframe in the simulation. The probabilistic algorithm leads to tolerance in the simulation. This risk minimizes the efficiency of the method. The experiment was conducted using qualitative data. If more industry-level quantitative tools were made available, the program could enhance the generalizability of the results.

## 7. Conclusions

An innovative feature of the proposed FL method is that it allows scene communication and generates standards. The predictive model with an FL network adds weight to public ledgers, which have more frequently voted for updates to the mathematical model. These highly connected ledgers, which belong to the data contributors, provide a solid consensus on the ability to predict fruit lifespan trends. This method assigns an internal reinforcement learning model to each ledger, and the percentage of adopted strategies can cover the existing market share of the problem to reflect the positive correlation of jointly simulating the decision-making environment under the same cornerstone. This interaction generates a highly influential model using blockchain technology.

The proposed prototype is based on blockchain technology to accommodate the features of decentralized governance and immutability and to align with the general trend of supply chain management in driving toward deploying distributed databases. The advantage of such an event-driven system architecture is that it involves more stakeholders at different stages of the process. With such decentralized governance, there is no unified authority in the system. Individual players cannot dominate or manipulate operations. In addition, scene applications provide a more flexible and scalable method for users to establish forecasting norms without middlemen. Using blockchain technology, critical parameters are stored in a public ledger. As the updated parameters are timestamped, they cannot be tempered by hackers. This implies that every user can read the data but cannot alter or reverse it. These records are permanently stored on the computers of the individual nodes. One critical feature in the development of AI policy is that the scene feature library is closely related to the scene consensus applied to the backbone technology of the double helix system. Finally, it produces scenarios that facilitate risk minimization and cost reduction of IoT devices used in the supply chain to derive a global optimal policy with the support of AI insight.

There are several potential directions for future development in blockchain-based federated learning. Some possible areas of focus include the following:

- Improving the efficiency and scalability of federated learning systems so that the systems can handle larger and more complex datasets.
- Investigating blockchain technology to enable new forms of decentralized AI model sharing and collaboration, such as creating “AI model marketplaces” where organizations can buy and sell access to anonymize the AI model *via* smart contracts.
- Researching ways to incentivize participation in federated learning systems, such as using digital currency-based rewards or other financial incentives.

## Data availability statement

The raw data supporting the conclusions of this article will be made available by the authors, without undue reservation.

## Author contributions

HC and KC: write. CL: experiment, program, and write. All authors contributed to the article and approved the submitted version.

## Footnotes

^1^Fresh mango Export Company and exporters in Indonesia. Tridge.com. Available online at: https://www.tridge.com/intelligences/mango/ID/export (accessed September 1, 2022).

^2^Helium roaming services. Helium.com. Available online at: https://docs.helium.com/use-the-network/roaming (accessed November 28, 2022).
